# Lessons learned: Shaping the evolution of veterinary specialty education

**DOI:** 10.1111/jvim.16936

**Published:** 2023-11-17

**Authors:** Jane E. Sykes

**Affiliations:** ^1^ Department of Medicine & Epidemiology, School of Veterinary Medicine University of California‐Davis Davis California USA

**Keywords:** accreditation, certification, competency, DEI, diversity, Flexner, graduate, internship, Millis, oversight, residency, specialization, wellness

## Abstract

In response to concerns regarding numerous complex issues facing the veterinary specialty profession, several organizations, including the American College of Veterinary Internal Medicine, have made a clarion call to the American Veterinary Medical Association to begin discussions surrounding the formation of an accrediting body for internships, residencies, and fellowships. A proposed name for such a body is the Accreditation Council on Graduate Veterinary Medical Education, in alignment with the Accreditation Council on Graduate Medical Education (ACGME); the term “graduate” refers to specialty education that occurs after the first 4 years of the MD or DVM degree. Although the structure and financing of graduate education differ between the human medical and veterinary professions, we can nevertheless learn much from the history of evolution of human medical specialization as we navigate the path ahead.

**Abbreviations**
AAMC: Association of American Medical Colleges
ACGME: Accreditation Council on Graduate Medical Education
ACGVME: Accreditation Council for Graduate Veterinary Medical Education
AMA: American Medical Association
CMEH: Council on Medical Education and Hospitals
CMSS: Council on Medical Specialty Societies
LCGME: Liaison Committee for Graduate Medical Education
RRC: Residency Review Committee

## 1 INTRODUCTION

“If we are to gain a grip on health care in the present, history can be a kindly, useful mentor.”

Rosemary A. Stevens (1997), American Medicine and the Public Interest: A History of Specialization.

Current estimates of dog and cat ownership in the United States^1^ indicate that there are over 1.5‐fold more client‐owned dogs and cats than there are people in the United Kingdom and Australia combined. According to the Tufts Equity in America survey, in 2022, 62% of US households had at least 1 pet^2^; the American Pet Products Association estimated that 66% of US households owned a pet.^1^ Animals are often considered household members, with the same expectations for healthcare quality and availability as exists for humans. As realized in the 1960s and 70s in human medicine, an explosion of knowledge and technological innovation has led to increased recognition of the importance of having a highly trained and effective specialty veterinary workforce to meet the needs of the animal‐owning public. In 2017, the Dean of the Faculty of Veterinary Medicine at the University of Calgary expressed concerns that crushing ever expanding content into the same 4‐year curriculum may be contributing to “reported increases in learning disabilities, stress, and anxiety among veterinary students,” and suggested options for lengthening the curriculum, including creation of graduate programs that allow students to pursue specific interests.^3^ Demand for veterinary specialty services is at an unprecedented high, and government support for growth of veterinary specialty medicine through expansion of the availability of training programs that hold both the educational and wellness needs of veterinary graduate trainees to high standards is urgently needed. The growth of the distributive model alone for training entry‐level veterinary graduates will not help efforts to train more specialists in academia, because the number and size of existing teaching hospitals are likely to expand at a rate that is woefully slow. Instead, we are likely to see growth of training opportunities in private specialty practices, which could vary considerably in their caseload, range of specialty offerings, expertise, experience training candidates, support for wellness and diversity matters, and the ability to offer structured training program components such as journal club and group case rounds. Without oversight, we are likely to see a similar large degree of variability in the quality of specialists that are trained; high‐quality veterinary specialists could be difficult for pet owners and referring veterinarians to identify, with an overall reduction in respect for the veterinary specialist.

In a 1974 article in the *New England Journal of Medicine*, the term *graduate medical education* was defined as “the training that a physician receives after graduation from medical school but before entry into practice. It encompasses internship, residency, and fellowship training and is the period during which specialization or subspecialization occurs.”^4^ Founded in 1981, the Accreditation Council for Graduate Medical Education (ACGME) is an independent, not‐for‐profit organization that evaluates and accredits physician graduate medical training programs in the United States, including internships, residencies, and fellowships; specialty college participation in the accreditation process is on a voluntary basis.^5^ Over 30 years after its founding, a crisis in the veterinary profession, as evidenced by the results of recent AAVMC and ACVIM residency wellbeing surveys that have identified widespread stress and lack of adequate mentorship for trainees, has stimulated a call for formation of a similar organization to ensure that graduate veterinary medical training programs (1) adhere to college specialty training requirements and (2) provide the necessary support for trainees to succeed.

In an article in the *Journal of the American Medical Association* titled “The Internship: Origins, Evolution, and Confusion,” read before the 1964 Annual Congress on Medical Education, James Campbell describes the birth of the medical internship, beginning in Europe as early as 1617, and the establishment of interns and residents in US hospitals in the late 1800s.^6^ Abysmal standards in undergraduate medical education in the United States then led the American Medical Association (AMA) to establish the permanent Council on Medical Education and Hospitals (CMEH; later named the Council on Medical Education) in 1904.^7^, ^8^

In 1905, Andrew Carnegie founded the Carnegie Foundation for the Advancement of Teaching. Carnegie was a Scottish‐born philanthropist who grew up in Pittsburgh. After selling Carnegie Steel Company to J. P. Morgan in 1901, Carnegie became the richest American, temporarily overtaking John D. Rockefeller.^9^ Concerned about the disorganized state of US medical education, the AMA engaged the Carnegie Foundation to initiate a review of the quality of medical school education across North America.^8^ The President of the Foundation, Henry Smith Pritchett (immediate past President of Massachusetts Institute of Technology) appointed the schoolteacher Abraham Flexner to work together with the Secretary of the CMEH, Dr. Nathan P. Colwell, to inspect and report on the quality of medical schools across North America (the “Flexner‐Colwell surveys”).^8^, ^10^, ^11^ Pritchett became familiar with Flexner after reading Flexner's 1908 book *The American College*, which described the deficiencies of the American college education system in comparison to German pedagogical models. This culminated in the landmark 363‐page Flexner report of 1910,^12^ which shaped current‐day American healthcare curricula and ultimately veterinary medical curricula as well.^7^, ^11^, ^13^, ^14^ Remarkably, a search of PubMed using the search terms “Flexner” and “veterinary” yielded only 12 results, none of which were about the Flexner report as it related to veterinary medical education; a search of the *Journal of the American Veterinary Medical Association* yielded only 2 letters to the editor and 2 commentaries that referred to Flexner's influence on veterinary medical curricula, published between 2012 and 2017.^3^, ^14^, ^15^, ^16^ Thus, it seems important that—for the first time—more history be provided here for readers of the *Journal of Veterinary Internal Medicine*. Although Flexner's work primarily addressed the core MD (“undergraduate”) curriculum, it subsequently became the basis for evolution of specialty education, another reason for expanding upon it here.

Abraham Flexner has been described as the man who made the greatest single contribution in history to the advancement of US medical education.^17^ Born to Eastern European Jewish immigrants in Louisville, Kentucky in 1866, Flexner was able to pursue an accelerated bachelor's degree at Johns Hopkins University after his 2 older brothers each obtained a medical degree at the University of Louisville. His oldest brother, Simon, became a successful academic pathologist under the supervision of William Welch at the Johns Hopkins School of Medicine, ultimately rising to become director of the Rockefeller Institute in New York.^17^ After leaving Hopkins, Abraham became a highly successful schoolteacher in Louisville. In pursuit of more intellectual stimulation, in 1904, he obtained a master's degree from Harvard and then spent time studying pedagogical methods in the United Kingdom and Europe, especially Germany. After a year in Berlin, he wrote *The American College*. His work on the Flexner report followed. Using Hopkins as the benchmark, Flexner described his findings following inspection and evaluation of 155 schools in 16 months—a dismal state of affairs—the majority of schools having low admissions standards, absent laboratory facilities, and inadequate opportunities for clinical experience.^12^ The Flexner report and parallel efforts from the AMA to set standards^8^ ultimately triggered the closure of up to 22% of American medical schools,^18^ and many schools of color, including 1 in Flexner's hometown, Louisville.^19^ Flexner then worked with William Welch, founding Dean at Hopkins and President of the AMA; William Osler, first chief of medicine at Hopkins; and Frederick Gates, an adviser to John D. Rockefeller (the so‐called “Hopkins Circle”) to build a science‐based, experiential curriculum that was the basis for modern day medical and veterinary education.^11^, ^13^ Of interest, Osler did not fully support Flexner's model, criticizing it for a lack of focus on the needs of the patient and the student; instead, the patient was seen to support the instructional needs of the professor.^11^, ^13^, ^20^ However, strong backing from the Carnegie and Rockefeller Foundation, together with Osler's move to Oxford at the time, crystallized Flexner and Welch's approach as the basis for current‐day medical education. By 1920, the United States overtook Germany as the leader in medical progress.^21^ Today, while the Flexner model remains held in high regard, concerns mirroring those of Osler's have led to adjustments in the medical (and veterinary) curricula to refocus on the patient and student experiences, with incorporation of instruction on doctoring skills, ethics, and social sciences.^11^

As knowledge grew, specialty training evolved. Two movements formed the basis of quality assurance: the “Certification” movement, whereby individuals who had achieved additional training were judged by selected peers; and the “Accreditation” movement, which worked to ensure quality in places that trained these individuals.^22^ On the certification side, the first program for education and recognition of specialists was developed by the University of Minnesota in 1915 in conjunction with the Mayo Clinic; the first certifying American Board was the American Board of Ophthalmology, established in 1917.^7^ Proliferation of specialty training programs then continued into the 1950s, each with its own certifying board. By the late 1950s, there were 18 American certifying boards, representing diverse fields such as neurologic surgery, ophthalmology, and urology.^7^

On the accreditation side, although the AMA's CMEH had recommended in 1905 that internships be included as part of the “ideal standard” for MD training, closure of medical schools after the Flexner report led to profound mismatches between the supply of undergraduates and availability of internship training programs.^6^ The CMEH conducted the first survey of internships in 1912; a list of hospitals approved for internships was first published in 1914.^8^ However, by 1927, only 11 medical schools required university‐approved internships for graduation. The recognition that major deficits existed in residency training programs led CMEH to publish the first list of hospitals approved for residency training in different specialty areas. The CMEH also developed basic standards for internship, residency, and fellowship training programs, entitled “Essentials of an Approved Internship” and “Essentials of Approved Residencies and Fellowships.”^6^, ^23^, ^24^ An approving body was also created within the CMEH, the Internship Review Committee.^6^

In 1952, the CMEH proposed to specialty boards that they establish similar residency review committees (RRCs), in contrast to the veterinary specialty residency training program committees that exist today, the RRCs were staffed and financially backed by the AMA.^24^ However, by 1962, ongoing concerns regarding the quality of internship and residency training programs led the AMA to commission an external review of these programs, which represented the first comprehensive review of medical education by the AMA since the Flexner–Colwell surveys. To conduct the review, the AMA formed the Citizens Commission on Graduate Medical Education, which was chaired by John S. Millis, a physicist from California who was at that time President of Western Reserve University in Cleveland, Ohio.^25^, ^26^, ^27^ When compared with Flexner, relatively little is written about Millis, but his contributions to medical education had comparable impact.^27^ In August 1966, after considering input from a wide range of stakeholders over a 3‐year period, the so‐called “Millis Commission Report” (entitled “The Graduate Education of Physicians”)^28^ was submitted to the AMA's Board of Trustees.^29^ Millis's report highlighted the fragmentation of graduate medical education, making the statement that “… any program of graduate medical education should be planned as a unified, progressive sequence,” condemning the dissociated internship and residency.^23^, ^28^ It was noted that although such dissociation might have been acceptable in the past, when many graduates went directly from internship to practice, in the 1960s, virtually every medical student voluntarily chose to complete an internship and 90% of those graduates continued onto residency training programs.^30^ Improved continuity of these training programs was imperative. As such, the Millis report recommended “… that the internship, as a separate and distinct portion of medical education, be abandoned and that the internship and residency years be combined into a single period of graduate medical education called a residency and planned as a unified whole.” Indeed, some boards were removing the internship requirement altogether and allowing graduates to enter specialty training directly from the final undergraduate year.^31^ Importantly, the Millis Report also called for the establishment of an independent, 10‐member Commission on Graduate Medical Education, which would have planning, coordinating, and reviewing authority over all graduate medical education programs.^28^, ^29^

In June of 1970, the AMA House of Delegates adopted 2 recommendations from the Council on Medical Education: (1) after July 1, 1971, new internship programs could be approved only after the application contained “convincing evidence that the internship and related residency years were organized and conducted as a unified and coordinated whole”; and (2) after July 1, 1975, no internship program could be approved that was “not integrated with residency training to form a unified program of graduate education.”^23^ In reaction to the controversy that this generated, it was emphasized that the decision had not been made without careful consideration of data and opinions from an enormous variety of stakeholders^23^; the same should be true as we consider how to structure improved oversight of internships in veterinary medicine. In a presentation on the future of the freestanding internship at the 1971 Congress on Medical Education, Max Michael Jr, an MD at the University of Florida, Gainesville, noted that the actions did not mean that internships or the term “internship” would be abolished, although he also suggested that the internship year be referred to as “the first postdoctoral year.”^23^ Should internships be dissolved into residency programs in veterinary medicine? If most veterinary graduates completed residencies, as was the case in human medicine at that time, this might be reasonable. However, it is unlikely this will ever be the case, given the relatively low return on financial investments in residency training that occurs in veterinary medicine. In addition, given the diversity of species interests that exist in veterinary medicine, internships offer veterinary graduates the ability to gain knowledge and skills for a wide range of career paths, without the need to commit to years of residency training and specialization. Perhaps of more relevance to our profession, Max Michael simultaneously underscored the need for collective (“corporate”) responsibility for graduate medical education by program directors and administrators within a training institution, as recommended in the Millis report:

We recommend that each teaching hospital organize its staff, through an educational council, a committee on graduate education, or some similar means, so as to make its programs of graduate medical education a corporate responsibility rather than the individual responsibilities of particular medical or surgical services of heads of services.^28^

In 1972, the AMA established the Liaison Committee for Graduate Medical Education (LCGME) to accredit programs, which was overseen by a Coordinating Council on Medical Education (CCME). The CCME also oversaw the Liaison Committee for Medical Education, which had been accrediting medical schools from 1942.^24^ The LCGME was comprised of representatives from the American Board of Medical Specialties, the Association of American Medical Colleges, the Council on Medical Specialty Societies, the American Hospital Association, the federal government, and the public. The inclusion of the American Hospital Association was important, given its role in formally recognizing hospitals as specialty training sites. The LCGME was expected to establish procedures for accreditation of an entire academic medical center's graduate medical education program.^32^ The Chair of the LCGME would rotate among the sponsoring organizations; support would consist of staffing from the AMA and money to support 50% of accrediting operations, the remaining coming from program fees and the 5 parent organizations.^24^ After its by‐laws were approved in 1975, the LCGME commenced its activities. The term “intern” was officially dropped by the Committee, instead referring to individuals in their first year of graduate medical education as “first year residents.” The RRCs were expected to continue to review programs and recommend accreditation status to the LCGME; after reviewing supporting information from an RRC, the LCGME (after consultation with the CCME) would then inform the program of its status. However, on examination of the procedures of the 23 RRCs that existed by the mid‐1970s, it became clear that their practices were dissociated and inconsistent, reasons for approval or disapproval of programs were not well documented, and many programs remained on probation for extended periods of time.^24^ Initially, attempts by the LCGME to define stricter protocols were not received well by the RRCs. Some progress was made once the RRCs were allowed to attend sessions of the LCGME and participate in deliberations, but RRC concerns persisted about the veto power of the CCME or the LCGME; the influence of the AMA on the RRCs, the CCME, and LCGME; appeal mechanisms; the delegation of authority; the role of RRC sponsors; and financial and staff independence.^24^, ^33^ In 1977, these concerns, as well as suggested approaches to streamline the accreditation process at reduced cost, were expressed in a letter to the AMA from William H. Muller Jr, the Chair of the Board of Regents of the American College of Surgeons.^33^ As a result of perceived inaction by the AMA, many of the suggested approaches were subsequently backed by the Council on Medical Specialty Societies.^33^ In 1980, the CCME separated itself from the LCGME and became a council, the Council for Medical Affairs, no longer having veto power.^33^ In 1981, the LCGME was replaced by the ACGME, for which the only policy guidelines were their bylaws and the “Essentials of Accredited Residencies in Graduate Medical Education.”^24^ The AMA subsidy was discontinued, and all costs of accreditation were passed on to the ACGME. The “Essentials of Accredited Residencies in Graduate Medical Education” was approved by all 5 ACGME parents and became effective in 1982.^24^ Of relevance to the current situation in veterinary specialty education, the section of this document that received most attention and delayed the approval process was the section addressing “the resident's agreement and responsibilities; benefits, including financial support, vacation, professional leave, and sick leave; the term of the residency; private privileges and other activities outside the educational program; the usual call schedule and schedule of assignments; and the guarantee of due process.”^8^ In 1982, there were 4573 accredited residency training programs in the United States.^34^

Today's ACGME has processes for accreditation of programs for 28 different specialties and many subspecialties.^35^ Program participation is voluntary, but programs that do not participate are not eligible for Medicare funding support.^5^ The ACGME website harbors links to training program requirements for each specialty (as dictated by the relevant specialty organization).^35^ Application for program accreditation must be initiated by an institution that has current institutional ACGME accreditation (a “Sponsoring Institution”). The ACGME describes sponsoring Institutions as “institutions that oversee and provide assurance for the quality of the working and learning environment in all their ACGME‐accredited programs. Each Sponsoring Institution must achieve and maintain institutional accreditation before it can sponsor 1 or more ACGME‐accredited programs.”^36^ Once the ACGME receives a specific program application from a Sponsoring Institution, a site visit may be performed before accreditation can be granted. Site visits are typically only conducted for core specialty training program applications (eg, internal medicine, radiology, surgery), and not for most subspecialty training program applications (the full list can be found at https://www.acgme.org/globalassets/pdfs/subspecialtieslist.pdf). Every 10 years, the ACGME revises a set of basic standards for training resident and fellow physicians, known as “Common Program Requirements.”^37^ As stated on their website, “These requirements set the context within the clinical learning environment for development of the skills, knowledge, and attitude necessary to take personal responsibility for the individual care of patients.” Addressing recruitment, the work environment, program personnel qualifications and behavior, basic educational program structural requirements, resident core competences, evaluation, professionalism, and well‐being, the 48‐page document is a critical resource for the veterinary profession as we continue to develop our own standards for veterinary specialty training.

While considerable differences exist between the structure and resources that support graduate medical education for physicians and those for veterinarians, problems identified for human graduate medical education in the 1970s—fragmentation of responsibility, variation in quality, inadequate response to public need for manpower with no central coordinating body, and inadequate in‐service evaluation of graduate clinicians—all exist currently within veterinary specialty training programs. These concerns have led to requests that the American Veterinary Medical Association begin conversations with stakeholders about the formation an oversight body resembling the ACGME, the Accreditation Council for Graduate Veterinary Medical Education (ACGVME).^38^ It may not be appropriate for all registered specialty organizations to participate in the accreditation process, and program participation could be voluntary, just as it is for the American Animal Hospital Association accreditation process. Evidence of accreditation, which could include site visits to ensure programs adhere to specialty college requirements, would send an important message to potential candidates seeking quality training in a good working environment, faculty and staff that wish to work in an institution that supports candidate educational and workplace experiences, and animal owners in need of high‐quality specialty patient care. Untapped opportunities exist to secure the funding and resources needed to establish such an accrediting body from private donors, industry, the government, and training institutions. We are now poised to craft the future of veterinary medical graduate education in a manner that reflects on the experiences of the medical colleagues that have walked the path ahead of us, to ensure that we secure the future of high‐quality specialty patient care for animal owners worldwide. We also have an opportunity to be inclusive in our approach, building on the progress that has been made through diversity, equity, and inclusion initiatives in both the medical and veterinary specialty fields, including those of the ACGME (https://www.acgme.org/initiatives/diversity-equity-and-inclusion/).^39^ Fortunately for us, there is ample documentation of all these experiences, and access to this information has never been easier than it is today. As Winston Churchill famously said in 1948, “*Those that fail to learn from history are doomed to repeat it*.”

## CONFLICT OF INTEREST DECLARATION

Author declares no conflict of interest.

## OFF‐LABEL ANTIMICROBIAL DECLARATION

Author declares no off‐label use of antimicrobials.

## INSTITUTIONAL ANIMAL CARE AND USE COMMITTEE (IACUC) OR OTHER APPROVAL DECLARATION

Author declares no IACUC or other approval was needed.

## HUMAN ETHICS APPROVAL DECLARATION

Author declares human ethics approval was not needed for this study.
